# Behavioural responses to influenza pandemics: What do we know?

**DOI:** 10.1371/currents.RRN1037

**Published:** 2009-09-09

**Authors:** Marta Balinska, Caterina Rizzo

## Abstract

The emergence of the novel A/H1N1 virus has made pandemic preparedness a crucial issue for public health worldwide. Although the epidemiological aspects of the three 20th century influenza pandemics have been widely investigated, little is known about population behaviour in a pandemic situation. Such knowledge is however critical, notably for predicting population compliance with non pharmaceutical interventions. This paper reviews the relevant scientific literature for the 1918-1920, 1957-1958, 1969-1969 influenza epidemics and the 2003 SARS outbreak. Although the evidence base of most non pharmaceutical interventions (NPIs) and personal protection measures is debated, it appears on the basis of past experience that NPIs implemented the most systematically, the earliest, and for the longest time could reduce overall mortality rates and spread out epidemic peaks. Adequate, transparent, and targeted communication on the part of public health authorities would be also of crucial importance in the event of a serious influenza pandemic.

## Introduction

The emergence of the novel A/H1N1 virus has made pandemic preparedness a crucial issue for public health worldwide. Knowledge of past pandemics is of substantial help when planning for a future one [1] and indeed the epidemiological aspects of the three 20th century influenza pandemics (1918-1920, 1957-1958, 1969-1969) have been investigated. But we know relatively little about population behaviour in serious pandemic situations. Furthermore, society has undergone major changes since 1918 (the scenario on which most pandemic plans have been based) and even since 1968.  In order to provide data on this subject, the European Union project FLUMODCONT [2] recently launched two research projects:

1. a population behavioural survey in four European countries (on-going), and
2. a literature search on population behavioural responses to past influenza pandemics and to the SARS outbreak. This paper reports and summarizes the results of the literature search.

## Search strategy and selection criteria

“Population behaviour” was defined as the response to and the social consequences of an influenza pandemic and SARS on the part of 1) the public health authorities, 2) health care workers, and 3) the general population. 

The present study is based on: 1) a systematic search in the American National Library of Medicine (PubMed) using the following MeSH terms: “Influenza AND disease outbreaks AND history”, “Influenza AND disease outbreaks AND public health response”, “Influenza AND disease outbreaks AND social behavior”, “Influenza AND disease outbreaks AND absenteeism”, “Influenza AND disease outbreaks AND travel”, “Cross-infection AND disease outbreaks AND influenza”, “SARS AND disease outbreaks AND history”, “SARS AND disease outbreaks AND social behavior”, “SARS AND disease outbreaks AND travel”, “ SARS AND disease outbreaks AND absenteeism”, “SARS AND disease outbreaks AND public health practice”, “Cross-infection AND disease outbreaks AND SARS”. Since there was no MESH term corresponding to school closure, we conducted a simple search combining “school closure” with both “influenza” and “SARS”; 2) a general Internet search using the terms: “Non pharmaceutical interventions AND pandemic planning”, “Absenteeism AND pandemic flu”, “Pandemic preparedness AND NGOs”, “Panic AND pandemic influenza”, “Pandemic AND historical data”, “Pandemic influenza history”, “Eyewitness accounts AND Spanish influenza”, “Pandemic history books”, “Population compliance AND pandemic influenza”, and 3) anecdotal information found in the Lancet, JAMA, the Times, and le Figaro. All literature that did not include direct or indirect elements relevant to behavioral response as defined above was excluded from the analysis.

Exclusive of anecdotal information and articles written in unknown foreign language (for the authors), a total of 124 relevant sources (105 journal articles and 19 Internet links) were located, covering the following topics: absenteeism, border screening and quarantine, community planning, disease and social class, disease spread, effects on the economy, ethical considerations, health seeking behavior, hospital preparedness, infection control in hospitals, long term effects of influenza, personal protection measures, psychological aspects of epidemics, role of health care workers, role of the media, role of pharmacists, school closure, stigma, surveillance and reporting. The majority of data related to the 1918-1920 pandemic. Almost no information was identified on “population behaviour” during the two subsequent pandemics. Information on the SARS outbreak concerned mostly infection containment. Finally, prospective studies in view of a future pandemic included surveys, modeling, analyses, and simulation exercises.

## Results

**1. 1918-1920**

1.1 Response of the public health authorities

In 1918, the public health authorities rapidly recognized the influenza outbreaks as a pandemic similar to that having occurred in 1889-1890 and consequently expected three epidemic waves: the first mild, the second virulent, and the third declining [3]. They knew the incubation period of influenza to be 2-3 days, foresaw problems of over-reporting (confusion between influenza and influenza-like-illness), and feared public panic [4]. In some countries (such as France and Great Britain), the medical establishment was confident that they could control the situation[5] [6], whereas in others (such as Germany and Italy), they were overwhelmed by pessimism and frustration [7] [8]. Experimental treatments were offered and tested and medical profiteering was not rare. In general, however, the reaction of physicians and nurses appears to have been selfless. Accounts of the time bear witness to their untiring efforts [9].

The prevention measures introduced (or non pharmaceutical interventions, henceforth NPIs) were largely the same as those considered today: school closure, restrictions on public gatherings, isolation and quarantine, health education, and personal hygiene (especially hand-washing and mask-wearing). The American Association of Public Health considered face masks the best prophylaxis after isolation, but recognized the difficulty of using them on a broad scale [10], and in Sydney it was noted that while face masks were compulsory, there were “many fewer” cases of influenza [11].

Regarding the effectiveness of NPIs, the most systematic study identified by the literature search examined 43 cities across the United States with markedly different incidence and mortality rates[12] [13]. Overall, those cities which implemented the largest range of NPIs (as listed above), the earliest, and the longest experienced the least mortality and were able to spread out epidemic peaks. Similarly, a model using historical data from 17 American cities found that those which introduced NPIs the soonest effectively slowed down the progression of the epidemic and reduced cumulative death rates by c. 50%; however, cities implementing NPIs during the first wave were at greater risk of mortality during the second [14].

NPIs were not successful in all countries. In Italy, a recent study found no difference in mortality rates between those cities which implemented NPIs and those that did not [15]. In Leipzig, it was felt that social restriction measures and recommendations for personal protection were of little avail in slowing the course of the epidemic [16]. While encouraging and/or enforcing NPIs, the public health authorities were not oblivious to their impact on the economy and, ultimately, on social welfare (notably through job and income loss). In Canada, the Ontario Board of Health had concluded - before the epidemic invaded the province – that reporting, isolation, and quarantine were “impracticable” [17]. The New York City public health authorities believed in the effectiveness of “rational prevention measures rigidly enforced”, but recognized the “many difficulties” involved [18]. Indeed, some went as far as to say that the harm brought about by NPIs may even have “outweighed their possible good effect”, in particular owing to the dislocation/paralysis of trade and loss of employment [19].

1.2 Consequences of the pandemic on the general population

Regarding the effectiveness of NPIs, one of the difficulties was public compliance. Compliance was seen to wane with time (when the preliminary wave of fear declined), for environmental reasons (keeping people indoors on hot nights) [20], for reasons of psychological stress due to isolation [21] or quite simply once they were no longer compulsory. Some governments did not re-impose social distancing measures during the second wave of the epidemic because of the major disruption they had caused [22].

Conflicting recommendations from various national and local authorities combined with widespread absenteeism and insufficient medical staff owing to military obligations (up to 50% in Great Britain) [23] can only have contributed to a sense of chaos and social disruption. Widespread panic has been documented for Australia [24] [25] and the United States, leading some “systematically” to abandon the sick [26]. Despite the media censorship imposed in warring countries, the French daily le Figaro reported in August 1918 that influenza was undoubtedly worse in France than anywhere else in Europe [27] and it was believed that the very survival of the French “nation and race” was at stake [28]. By contrast, in Great Britain readers of the Times were told that, whereas over 4000 people had perished in the last week of October 1918, they must “see things in perspective, as a stout heart is a great safeguard these days” [29].

Several studies have cast doubt on the generalized belief that the 1918 pandemic struck evenly across society (regardless of wealth or ethnicity) if not across the world. Convincing evidence that poorer social groups suffered greater mortality has been provided for the United States [30] and Norway [31], and in South Africa it was reported at the time that the “local population” fared far worse than the Europeans in terms both of influenza incidence and mortality [32] [33]. In Italy, higher mortality rates were associated with type of profession [34], whereas in Great Britain no correlation was found between any kind of social status and mortality; only pregnant women died in greater numbers [35]. Some experts believed that above all malnourishment, lack of adequate clothing, and poor housing (such as in India) explained higher death rates [36], while others pointed out that neutral and relatively prosperous Switzerland fared worse than war-torn Germany [37]. The most noted sequelae of the 1918-1920 pandemic was so-called “chronic nervous influenza” [38] [39] [40], and depression due to long convalescence. A “harvesting effect” was noted in France, especially for young people suffering from tuberculosis and cardiovascular disease [41].

**2. 1957-1958, 1968-1969**

A total of three relevant articles was identified for the two other 20^th^ century pandemics. One study conducted in the United States found that in 1957 influenza strains had spread mostly through schools, contrary to 1968 [42]. In Singapore, between 10% and 30% absenteeism in commercial firms is believed to have had a severe impact of the economy in 1957-1958 [43].****

**3. Severe Acute Respiratory Syndrome (SARS)**

The containment of SARS, both on the national and international levels, was exemplary. Nevertheless, a certain number of hindrances and complexities were subsequently identified which can be of use when planning for an influenza pandemic.

3.1 Response of the public health authorities

One of the first measures to be taken to limit the outbreak was the screening of persons returning from infected countries. In Canada, the health authorities distributed leaflets to passengers in several languages, introduced compulsory screening (interviews), and installed infrathermal scanning machines. But, according to one author, screening effectiveness was necessarily limited by the fact that travel time to Canada from any point in the world is less than twenty-four hours, thus making the detection of a case during incubation highly unlikely [44]. On the other hand, a mathematical model seeking to evaluate measures taken to reduce the international spread of SARS found that border screening had been about 10% effective, whereas the distribution of information had played only a minor role. The authors of this study argued that an effectiveness of even 10% could provide up to a week’s delay in spread [45].

Quarantine was also widely used. In Taiwan, 150,000 people were quarantined of whom only 24 were subsequently found to have laboratory-confirmed SARS. Quarantine proved to be effective in improving onset-to-diagnosis time and limiting infection [46]. In Singapore, the military was involved in enforcing home quarantine [47]. Long historical experience has shown quarantine to be one of the only measures that can be taken in face of a lethal outbreak, but it is usually painful for those involved. Depression and post-traumatic stress disorders are known to affect certain quarantined people, prompting suggestions that in such situations emotional support be provided by public health authorities [48]. A web-based survey of 129 people quarantined for SARS in Taiwan indicated that only 68% of respondents understood the reasons for quarantine (not related to educational level), that a mere 30% felt they had received adequate information, and that adherence to control measures was suboptimal [49]. A major problem associated with quarantine is potential financial hardship especially for disadvantaged socio-economic groups, and the effects on the economy owing to absenteeism [50].

In Hong Kong, mass education regarding SARS appears to have been relatively effective  [51]and in Singapore, information and education was regularly delivered through press conferences, public talks, hotlines, and websites [52]. A survey conducted on nearly 600 people attending polyclinics found that poor awareness of SARS was associated with older age, blue collar workers, housewives, and those living in three- or fewer-room flats. After logistic regression, the most significant predictor of poor awareness of the disease was educational level [53]. According to another study, women, 30-49 year-olds, and those with primary education only experienced the greatest anxiety [54].

3.2 SARS and health care workers

Most health care workers (HCWs) involved in the treatment of SARS patients found themselves in a totally novel situation: their job had suddenly become potentially life-threatening and many were cut off from their usual social environment (either due to their own isolation or fear of contagion on the part of friends and family). The compliance of HCWs was critical in the effort to prevent nosocomial spread and “leakage” of infection back into the general population. Beyond the actual hospital building characteristics (e.g. self-contained emergency room), the importance of developing and adhering to infection control programmes has been widely stressed[55] [56] [57]. Personal and patient protection could be problematic: two surveys conducted on HCWs and medical students in Hong Kong revealed that hand-washing was recognized as being effective, but that compliance was low. Reasons included suboptimal promotion (by hospital management) of prevention measures and a greater preoccupation with self-protection than with patient protection [58]. At the French Hospital in Hanoi, precautionary behaviour on the part of HCWs was seen to increase as the outbreak progressed [59]. A large study of HCWs (n = 14,554) in five different settings in Singapore concluded that communication programmes capable of reaching all levels of HCWs were necessary to address the large number of factors determining the appropriate use of protective equipment [60]. Indeed in Vietnam, a retrospective survey found that compared to HCWs who always wore a mask, those who never did had a 12.6-fold higher risk of developing SARS [61]. Analysis of the SARS outbreak in Canada has underlined the importance not only of personal protection but also of moral support for HCWs and patients exposed to a potentially lethal infection. Given the shortage of staff, nurses were obliged to work up to 2 to 3 shifts in a row. Yet despite their key role in caring for patients, they were given “a minimal voice in drafting internal policies and procedures as to how to deal with the SARS epidemic” [62]. One author commented on the necessity for hospital psychiatric departments to develop epidemic response plans in conjunction with hospital administration, given the psychological management necessary for both patients and staff during a lethal outbreak [63]. Some have concluded that the greatest limitation in the response to the SARS outbreak was the absence of coordinated leadership, stressing the importance of communication between the critical care community, hospital management, government, and public health officials [64].

**4. Current surveys and modeling in view of a “future” influenza pandemic**

4.1 Possible responses on the part of public health authorities

A study of NPIs based on the recent literature and “expert opinion” concluded that there are very few high quality studies to inform the evidence base for NPIs (Table 1). It is generally accepted that no one NPI implemented on its own would be effective in lessening the spread of disease in a pandemic situation, with the possible exception of early isolation of cases and voluntary quarantine [65] [66]. Modeling tends to show however that the *concomitant* use of NPIs could be very helpful in delaying the temporal effects of an epidemic by reducing peak attack rates and allowing time for preventive and therapeutic interventions as well as reducing the burden on health care systems [67], providing the basic reproductive number, Ro, remained below 2 [68]. 

***Table 1. NPIs recommended on the basis of a review of the literature and expert meeting (according to*** [69])

**Table:** 

**Measure**	**Recom.** **Yes/No**	**Evidence-base**	**Commentaries/** **Advice**	**Problems**
Hand hygiene	Yes	Widely supported by literature	Antimicrobials have not been shown to be better than soap	Compliance: washing must be rigorous and routine throughout all phases of pandemic Supply and cost Habit of handshaking
Respiratory etiquette	Yes	Widely supported by literature	Need for public education	Need to sneeze into sleeve or tissue and not hand; no spitting.
Surgical masks for HCWs	Yes	Probably offers less protection than N95 respirators	Both surgical masks and N95 respirators should be available for HCWs	
N95 respirators for HCWs	Yes	Designed to stop up to 95% of small airborne particles	Both surgical masks and N95 respirators should be available for HCWs	
Isolation	Yes	Efficacy unsure given that viral shedding begins before symptoms	Probably useful if outbreak is short. Voluntary, home self-isolation recommended for all phases of pandemic.	Disagreement as to mandatory isolation.
Masks and respirators for general public	No	Relative roles of droplet vs aerosol transmission not established		Supply Competency and adherence
Social distancing measures, school closure	No	Efficacy not demonstrated		Legal and ethical problems
Screening of travelers	Yes/No	Efficacy not demonstrated		Disagreement regarding usefulness and justification.

There is no clear consensus regarding the usefulness of screening and travel restrictions in an influenza pandemic situation. The 1918-1920 pandemic demonstrated that even in the absence of air travel (although military and civilian movements were occurring on a large scale), influenza spread rapidly, as it did in 1957 and in 1968, when the volume of air traffic was not comparable to what it is today. And in 1968, it appears that influenza spread quickly and extensively by international air travel [70]. Nevertheless, it has been pointed out that most models investigating this issue prospectively assume that people banned from air travel would drop their trip altogether, whereas this is not necessarily true [71]. In any case, most stochastic models have shown that the international spread of influenza would be very little affected even by the most rigid travel restrictions. One study suggested that border restrictions could delay spread by 2-3 weeks, but only if effectiveness were over 99% [72].

Border screening is generally not considered to be an efficacious prevention measure, given influenza incubation periods, the need for highly sensitive tests, and the expense of the procedure, although some feel that it might “reassure” public opinion in a pandemic situation [73]. 

School closure, on the other hand, is one of the first containment measures advocated by national pandemic preparedness plans, and has been employed in certain countries in the wake of the influenza outbreaks in Mexico and the United States. The main arguments in favour of school closure are based on the assumption that children are major influenza spreaders. Schoolchildren have indeed been shown to be important vectors of seasonal influenza [74] [75]. One individual based model assuming no vaccination, use of antiviral drugs or pre-existing herd immunity found that the size of an outbreak could be limited by an estimated 10% if schools were closed as soon as more than 50 cases were detected in the community [76]. A recent study developed a transmission dynamics model using data from 1957. It found that the impact of school closure would depend on 1) contact patterns in the population 2) how school closure effects these patterns 3) the interplay of different age groups, and 4) the stage at which the measure was implemented. The authors concluded that in a future pandemic the closure of schools and nurseries would probably reduce the size of the epidemic “only by a small amount” [77]. But other models have determined that school closure during a pandemic could reduce the number of cumulative cases by 13-17% and peak attack rates by 39-45% [78]. All studies stress that in order to be effective school closure would have to be accompanied by other prevention measures. Those who argue against school closure stress its social consequences (in particular for low-income groups), economic costs, and the lack of firm evidence regarding its efficacy [79] [80] [81]. 

4.2 Possible consequences of a “future” pandemic on the general population

A distinction should be made between “behavioral” and “emotional” implications in a pandemic situation. Population behavioral reactions include panic, non compliance, resistance to travel restrictions, breaking of quarantine and isolation, and civil unrest, whereas emotional responses to a severe epidemic could lead to increased anxiety, depression, suicide rates, traumatic stress reactions, and complicated grief [82] [83].

Regarding population behaviour, a nation-wide survey conducted in the United States in 2006 revealed that only 41% of the population knew what “pandemic” meant, 33% had heard of it without understanding the concept, and 25% had never heard it. A surprising 94% of the population stated that they would be able to stay home for 7-10 days if ill themselves, and 85% - if a household member were sick. 85% also believed that they could keep their child from attending public gatherings. But high percentages of single adults, chronically ill persons, low income groups, and ethnic minorities stated that they would have no one to look after them in case of illness [84].  As for most transmissible diseases, poor and marginalized groups would in all probability be both more susceptible to disease and potentially greater spreaders of infection [85]. 

Absenteeism due to childcare and personal illness is the main preoccupation when considering the economic and social consequences of a pandemic. Various studies have found that anywhere between 25% and 50% of staff would be absent from work at one point in time during a pandemic influenza outbreak, but not all the time [86]. According to a recent survey in the United Kingdom, an estimated 16% of the workforce would stay home in case of school closure. Absenteeism would vary considerably according to occupation (those who are able to work from home or who enjoy sufficient resources to forgo income temporarily) [87]. A national survey in the United States found that 86% of families where at least one adult was employed would be able arrange for childcare for up to 3 months, but 57% [88]recognized that they would have serious financial difficulties if they had to stay home for over one month. In Australia, it is believed that 38% of the workforce might be absent from work owing to childcare, but that 73% of these would be able to work from home [89].

Predicting absenteeism levels of HCWs is clearly a crucial issue for pandemic preparedness. A UK survey found that the profession likely to be the worst affected by school closure would be precisely the health sector, given the impossibility for HCWs to work from home and the fact that the majority of this workforce is made up of women (the main caregivers in society). According to this survey, absenteeism for childcare and due to personal illness at the peak of the epidemic could lead to a 45% reduction in HCWs' attendance [90]. In Georgia, a cross-sectional study suggested a 23% absenteeism rate (mainly among women and nurses) if the incidence of pandemic influenza for HCWs was twice that observed for the general population [91]. A German survey conducted at the time of the SARS outbreak found that 28% of physicians and 1^st^ year medical students considered it “professionally acceptable” for HCWs to abandon their workplace during an influenza pandemic and a further 19% were “unsure” [92]. The willingness of paramedics to continue working in the event of a smallpox outbreak has also been examined: reporting to work was associated with younger age, male sex, single status, and no children [93]. 

The financial sector has carried out various simulation exercises in order to address its capability to cope with a pandemic [94] [95] and some of the main conclusions are presented in Table 2. With regard to the impact of a pandemic on freight transportation, even if absenteeism were of short duration (a few weeks) its impact would be felt well after the end of the epidemic [96]. As regards consumer activities, decrease would probably be influenced more by perceived risk than by actual attack rates, and over-demand would vary according to good service [97]. 

***Table 2. Main lessons learned during a simulation exercise concerning a serious pandemic influenza outbreak and business continuity (according to [94] ***)

**Table:** 

1. Response should be both stratified and opportunistic 2. Emphasis must be put as much on people as on the virus 3. It is likely that by day 28 all systems will have fallen apart 4. Engaging business in pandemic preparedness plans is crucial 5. Business must learn to work with media 6. Government, business, and organizations must be encouraged to work together 7. Martial law would be an end but not a means 8. A pandemic situation needs to be defined locally, as well as how government and business should work together in such a situation 9. A dashboard would be needed to track influenza spread around the world 10. The military must be involved in control measures.

## Discussion

The importance of transparency on the part of public authorities has been clearly illustrated by a series of health “crises” in Europe over the past quarter of a century (Chernobyl, HIV-contaminated blood banks, “mad cow” disease, alleged vaccine side effects, etc.). Cleary, public trust has been influenced over the years by how authorities deal with health problems and emergencies and, in the event of a serious influenza pandemic, appropriate communication tailored to specific groups will be critical to disease control (Table 3). If we are to prepare for the worst while hoping for the best, the 1918 experience should be considered when planning for a “future” pandemic. Major changes since the deadly 1918 pandemic include our capacity to detect cases rapidly, improved health care, access to care, targeted pharmaceuticals, and overall coordination. Yet we still face major difficulties in predicting the transmission dynamics of influenza, its local impact, and population compliance with prophylactic measures. Today, as then, the authorities will seek by various means to mitigate panic in the interest of disease control. Today, as then, shortages of HCWs – for a variety of reasons – are to be expected. Today, as then, business profiteering could occur, especially on the web (sales of “falsified” pharmaceuticals, protection gear, and vaccines as well as “miracle” and “natural” remedies). Public behaviour is likely to be similar in some respects (waning compliance with prevention measures as fear declines), but it is difficult to determine the respective effects on the population of the on-going “war effort” (in 1918) and a heightened perception of personal health and exposure to risk (today). Suboptimal adherence to prevention measures as a function of risk perception has been noted, or at least discussed, with regard to diseases such as HIV-AIDS [98] or Chikungunya [99]. Other major differences between 1918 and today include legal interpretations of privacy and civil and constitutional rights (for example, issues related to mass vaccination or post-pandemic law suits regarding resource allocation and alleged or real vaccine adverse events), but also improved base-line understanding of health messages. Finally, probably the single most important difference between today and then is access to information via the media and the Internet. In the 21^st^ century, people can find out what measures are being taken in other countries and this could lead to increased public demand, warranted or unwarranted criticism of public health policies, and conflicting perceptions of “fairness” of interventions and resource allocation. 

***Table 3. Ethical issues and possible responses (according to [94])***

**Table:** 

**Problems**	**Suggested solutions**
Rationing for essential prophylactic measures and resources (food, water, petrol…)	Priority should be given to: First responders, pregnant women and young children, young people (life years saved), “super vectors”
HCW attendance (in absence of personal illness or caring for family member)	Develop official ethical guidelines on balancing public needs and personal risk
Decision-making in absence of “evidence-based” information	Decisions/actions must be well communicated and perceived to be “fair”.
“Social degeneration” (absenteeism, chaos, other disease outbreaks, anxiety, looting…)	Industry, business, and organization preparedness, individual household preparedness, involvement of military, mental health care resources…

In the time elapsing before a targeted vaccine can be developed, produced, and distributed NPIs will be critical parameters of disease control, especially if antiviral stocks are insufficient and/or resistance to these drugs occurs. Surprisingly little evidence exists to guide our decisions in this area, with the probable sole exception of self-isolation. One of the reasons why it is difficult to determine the efficacy of NPIs is our as yet incomplete knowledge of influenza spread. In particular, it is not clear whether the majority of transmission occurs before the onset of symptoms or after, and relatively little is known regarding the seasonal drivers of transmission [100].  Experts during the 1918-1920 pandemic recognized precisely the same gaps in knowledge: “*It is not our isolation that has failed,”* the Lancet reported in 1920,* “ but that our limited knowledge of the causes of [influenza] spread is as yet unable to supplement it by suitable actions in other directions”* [101].

## Conclusion

Our review contains several limitations. To begin with, we searched systematically only one database (PubMed), used MeSH terms (which are not necessarily exhaustive) and by definition our findings suffered from English-language publication bias. We did not have access to unpublished material, such as doctoral dissertations, public or private surveys. We were not able to consider the economic consequences of NPIs in a future pandemic scenario, since data was lacking. Finally, we did not compare national pandemic preparedness plans (in developed countries) with regard to expected population behaviour and NPIs. A thorough search and comparison of a selection of the most important daily newspapers for the epidemics considered would also undoubtedly have revealed precious information, at least regarding media-perceived public reaction. Despite these gaps in information, we believe that this review is one of the first to gather and analyze data on population behaviour in pandemic or serious outbreak scenarios over the past hundred years.

What practical lessons can be drawn on the basis of this literature search?

From a historical perspective, four main conclusions stand out. One, non pharmaceutical measures (school closure, restrictions on public gatherings, isolation and quarantine, public and HCW education) – regardless of their “evidence base” – implemented the most systematically and for the longest time would *probably* have the greatest prevention effect in the absence of an efficacious vaccine. Two, population adherence to public health measures and messages might well be high during the initial phase of an epidemic perceived as dangerous, but then decrease with time. Three, enhancement of infection control programmes in hospitals is necessary for preparedness. Finally, adequate and transparent information on the part of heath care authorities and in collaboration with the media, business, and organizations will be paramount for disease control and containment.

## Competing Interests 

The authors declare no conflict of interest

## Access to data 

The corresponding author declares she has had full access to the data used for this literature search.

## Funding 

This literature search was funded by the FLUMODCONT project ([2]).

## Acknowledgements 

We grateful to Antonella Latanzi (Istituto Superiore di Sanità for her kind assistance with bibliographical sources. We also thank Silvia Declich (Istituto Superiore di sanità) for her support in conducting this study and Cécile Viboud (National Institutes of Health) for her technical guidance.
